# The COVID-19 Host Genetics Initiative, a global initiative to elucidate the role of host genetic factors in susceptibility and severity of the SARS-CoV-2 virus pandemic

**DOI:** 10.1038/s41431-020-0636-6

**Published:** 2020-05-13

**Authors:** The COVID-19 Host Genetics Initiative

**Affiliations:** 1 Institute for Molecular Medicine Finland, University of Helsinki, Helsinki, Finland; 2 Analytical and Translational Genetic Unit, Massachusetts General Hospital, Harvard Medical School, Boston, MA USA

**Keywords:** Genetics research, Social sciences

## Introduction

The COVID-19 pandemic is a global crisis creating severe disruptions across the economy and health system. Insights into how to better understand and treat COVID-19 are desperately needed.

Early studies have focused on the clinical characteristics [1–3], epidemiology [1, 4, 5], and genomic characterization [6–8] of SARS-CoV-2 infection. These studies have also highlighted the value and importance of transparent data sharing across countries, which have enabled the live tracking of the disease widespread worldwide [9, 10]. The role of host genetics in impacting susceptibility and severity of COVID-19 has been less studied. Previous work has supported the role of human leukocyte antigen (HLA) in susceptibility [11] and severity [12] for several viral infections. Moreover, a synonymous variant in the IFN-induced transmembrane protein-3 gene has been reported to cause severe clinical outcomes in patients infected with H7N9 and H1N1 influenza viruses [13, 14], although results did not reach established *P* value thresholds (*P* < 5 × 10^−8^). In addition, candidate variant studies have suggested host factors that are critical for severe disease in other coronavirus infections, such as infections due to the related SARS-CoV [15].

Given the importance and urgency of exploring the role of the host genome in conjunction with COVID-19 clinical and genomic variability, and the recognition that this can only be achieved with the combined effort of the scientific community, we launched the ‘*COVID-19 Host Genetics Initiative*’. This initiative brings together the human genetics community to generate, share, and analyze data to learn the genetic determinants of COVID-19 susceptibility, severity, and outcomes. Such discoveries could help to identify individuals at unusually high or low risk, generate hypotheses for drug repurposing, and contribute to global knowledge of the biology of SARS-CoV-2 infection and disease. The initiative has three main goals:

1. Provide an environment to foster the sharing of resources to facilitate COVID-19 host genetics research (e.g., protocols, questionnaires).
2. Organize analytical activities across studies to identify genetic determinants of COVID-19 susceptibility and severity.
3. Provide a platform to share the results from such activities, as well as the individual-level data where possible, to benefit the broader scientific community.

### Approach

The COVID-19 host genetics initiative is a bottom-up initiative with a flexible, decentralized structure that is based on the following collaborative principles:

1. Collaborate in an environment of honesty, fairness, and trust
2. Promote early-career researchers
3. Respect other groups’ data
4. Operate transparently with a goal of no surprises
5. Seek permission from each group to use results prior to public release
6. Do not share another group’s results with other parties without permission
7. The initiative should not inhibit any work being done within any individual studies (or between pairs of studies).

Studies that are interested in joining the initiative can register via the website^1^. We can categorize the participating studies in two main groups. Retrospective collections are typically biobanks with existing significant genetic data and active connections to health systems. In these studies, there is the opportunity to opportunistically and rapidly develop a genetic study on susceptibility and severity. For example, in Finland with the national network of biobanks covering each hospital district, it is possible to acquire almost ‘real-time’ updates on COVID-19 status of individuals that are already part of the FinnGen study^2^. This group of studies is already connected and loosely structured via other initiatives such as the Global Biobank Meta-analysis Initiative^3^.

The second group of studies includes prospective collection that have recently started to directly consent incoming COVID-19 patients. More than just the critical jump in scale for studying progression, severity, and outcomes, these studies bring important additional opportunities not only for deeper DNA studies, but potentially informative viral and antibody profiling and epitope mapping experiments which can be implemented in many sites with relatively small blood/plasma requirements.

### Data sharing

We expect that a sizable fraction of the studies will be able to share individual-level data. Genetics and clinical data are submitted to the European Genome-phenome Archive (EGA) under controlled access, and this is coordinated with viral sequence deposition efforts and coordination of other biomolecular data with EU, EOSC, ELIXIR, and other institutions across the globe. Alternatively, studies are able to share summary statistics, which will be directly made available on the website and via the GWAS catalog [16].

The majority of the planning, discussion, and exchange of information between the participants study, analysts, and clinicians is done on a dedicated Slack workspace with the support of the International Common Disease Alliance (ICDA)^4^.

### Phenotype and analysis

The initiative aims to support widespread sharing of data and knowledge across participants groups. Groups can connect and initiate collaborations focused on specific phenotypes. Few analyses that can benefit from maximal sample size are centralized. The primary analysis focuses on COVID-19 disease severity. There are challenges in defining COVID-19 severity across multiple studies and healthcare systems. We used a pragmatic approach which considers the use of invasive and noninvasive ventilation as an index of severity. The advantages of this approach is the possibility to easily retrieve this information from electronic health records and the widespread use of these procedures across healthcares. Studies that have collected detailed clinical information can perform secondary analyses using continuous markers of disease severity such as maximum respiratory rate during hospitalization or prior to invasive respiratory support.

Bioinformatic and statistical analysis will consider data generated from GWAS array, exome and genome sequencing, leveraging the impact of both common and rare variants. Key analysis will take into account differences between sexes, ancestries, and date of sample collection. The latter aspect is important to consider given the rapid changes in population screening procedures and hospital capacity with consequent impact on the severity of patients included in different studies.

Given the importance of the HLA genes system for the etiology of infectious diseases and autoimmune disorders, we will impute classical HLA alleles and the corresponding amino acid sequences. COVID indiscriminately affects populations from all around the world, and HLA variation is specific to different populations. Hence, we propose using a multiethnic HLA reference panel constructed using deep-coverage whole-genome sequencing data from 21,546 individuals of five different populations: European, African, Latino, Asian, and South Asian. This reference panel will capture much of the HLA variation around the world. This will allow to test each HLA allele and also each of the amino acid site position within HLA genes to assess if they explain COVID risk.

### Participant studies

**Fig. 1:**
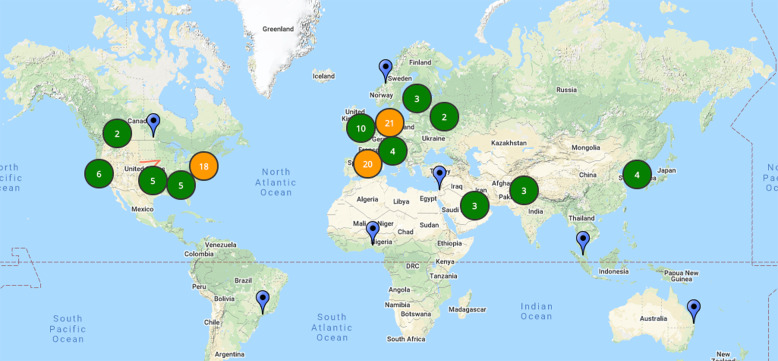
Map of the studies registered to the initiative by 13th of April 2020. The map report aggregate counts of studies registered to the COVID-19 Host Genetics Initiative.

At time of writing 105 studies have joined the initiative, and participation is still expanding. The majority of studies are conducted in Europe (55%) and the US (28%), amongst which the United Kingdom (10%) and Italy (9%) are the largest. However there are also participants from Asia (Republic of Korea and Malaysia), Australia, the Middle-East (Kuwait, Pakistan, and Qatar), and Africa (Nigeria); Fig. 1, an updated list is available on the website^5^. Most studies (71%) have initiated a new prospective collection, 27% have done that on top of existing retrospective collections. Array-based genotyping is the most common approach, considered by 69% of the participant studies, while exome and genome sequencing are less common, (29%). Antibody and immune profiling are the two most common additional assays that are reported by the contributing studies.

## Conclusion

We initiated a global effort to study the relationship between host genome and SARS-CoV-2 infection. Our approach is inclusive, decentralized, and transparent. While providing novel scientific insights remains a priority of the initiative, we equally value the creation of an infrastructure that facilitates communication between studies with similar scientific goals. We expect the *COVID-19 host genetics initiative* to substantially contribute to the understanding of the variability of COVID-19 susceptibility, severity, and outcomes in the population within the next few months.

## Footnotes

1 https://www.covid19hg.org/register/

2 https://www.finngen.fi/en

3 https://www.globalbiobankmeta.org/

4 https://www.icda.bio/

5 https://www.covid19hg.org/partners/
